# Supplementary material for: USP50 suppresses alternative RecQ helicase use and deleterious DNA2 activity during replication
Source: Nat Commun. 2024 Sep 16;15:8102. doi: 10.1038/s41467-024-52250-4 (PMC11405836; doi:10.1038/s41467-024-52250-4)
Supplement: Supplementary file 1 — Supplementary Information [file 41467_2024_52250_MOESM1_ESM.pdf]

Supplementary Figure 1.

```
CLUSTAL W (1.83) multiple sequence alignment based on NP_987090.2/Q70EL3

Ccow_USP50             MWFHEMASQRSFPED--DFSIYYV-LSECADH-YDPCPVNEA-EENQPHL
alpaca_USP50           -----MASRQPLPAD--DFGIYYV-LAEC-DH-YDPFPANEA-DENQPHF
anole_lizard_USP50     -----MASKSTKMPD--DFNIYYL-LKGVLPLETSEEPMDESRDETNPQY
bushbaby_USP50         -----------------SFSVDQH-SAECTDN-YDPLPVNEP-DESQPHF
cat_USP50              --------------------------AECTDH-YDSSPVKEV-EENQPHS
chimpanzee_USP50       -----MTSQPSLPAD--DFGIYHV-LAECTDY-YDTLPVKEA-DGNQPHF
dog_USP50              F--TKMTSQRPLPAD--DFSIYYV-LAECTDP-PDAPALAEL-RASPPRR
dolphin_USP50          -----MASQQPFTAD--DFGIYCV-LTECTDH-YDPFPVNEA-DENQPHF
elephant_USP50         S--IKMTSQRPLPVD--DFGIYYV-LAECTDY-YDTIPVNEV-DEDQPHF
fugu_USP50             --------------------------------------------------
guinea_pig_USP50       -----PSSEH-------SLSAHPH-STERTEL-PDPLPARED-PARPPNP
human_USP50            -----MTSQPSLPAD--DFDIYHV-LAECTDY-YDTLPVKEA-DGNQPHF
medaka_USP50           -----MNV------------------------HF--VPTS--QKEKKCRR
megabat_USP50          -----MTSERPLPAD--DFGIYYV-LAECTDH-YDTFPAKEA-DENQPHF
microbat_USP50         F--TNMTSQRPSPGD--DFGIYYV-LAECADH-CRGSPVHP--GGNQPHL
mouse_USP50            MCFIDMASHRPVPAD--DFGAYYN-LAECAD--YDSLPE----SKTQPHF
opossum_USP50          -----------------------------------------S-NMAQQHS
platypus_USP50         ----------------------------------------------GARA
rabbit_USP50           S--TTMTSQRSFPAD--DFGIYYV-LAECTDH-YDTLPVNEA-DKNQPHF
rat_USP50              VCYRNMASHRPFPAD--DFGAYYI-LAECAD--YDSLPE----SETQPHF
stickleback_USP50      --------------------------------------------------
tasmanian_devil_USP50  F--IERSCCHPAPQNHLSFSLWHKHSTECTDY-SDTSSVKES-SMAQQHS
tetraodon_USP50        --------------------------------------------------
wallaby_USP50          -----MA---QMPPD--DFKIYYV-LTECTDY-SDTSSVKES-SMAQQHS


Ccow_USP50             QGVTGLRNLGNTCYMNAILQCLCSISPLVEYFLSGKYINALQ--------
alpaca_USP50           QGVTGLRNLGNTCYMNAILQCLCSISPLVEYFLSGKYITALQN---F---
anole_lizard_USP50     QGLTGLRNLGNTCYMNAVIQCLSSVSPLVEYFLSGKYTAALD--------
bushbaby_USP50         QGVTGLRNLGNTCYMNAILQCLCSISPLVEYFLSGKYMTALQ--------
cat_USP50              QGVTGLRNLGNTCYMNAILQCLCSISPLAEYFLSGKYITALQN---F---
chimpanzee_USP50       QGVTGLWNLGNTCCVNAILQCLCSILPLVEYFLTGKYITALQ--------
dog_USP50              QGVTGLRNLGNTCYMNAILQCLCSVSPLVEYFVSGKYITALQ--------
dolphin_USP50          QGVTGLQNLGNTCYMNAILQCLCSISPLVEYFLSGKYITALQN---F---
elephant_USP50         QGVTGLRNLGNTCYMNAILQCLCSISPLVEYFLSGKYITALQ--------
fugu_USP50             ----GLDNSGNSCYLNAVLQCLCSTVPLVEYLLNQDTQAELA--------
guinea_pig_USP50       PGVAGLRNLGNTCYMNAVLQCLASVPPLAQYFRSGQYVTALR--------
human_USP50            QGVTGLWNLGNTCCVNAISQCLCSILPLVEYFLTGKYITALQ--------
medaka_USP50           PGVCGLENSGNSCYLNAVLQCLCATVPLVEQLLHWDTRKGLARYHHFILN
megabat_USP50          QGVTGLRNLGNTCYMNAILQCLCSISPLVEYFLSGKYITALQN---F---
microbat_USP50         QGVTGLRNLGNTCYMNAILQCLCSISPLVEYFLSGKYITALH--------
mouse_USP50            QGVTGLRNLGNTCYMNAILQCLCSVSPLVEYFLSGKYITALK--------
opossum_USP50          QGVTGLRNLGNTCYMNAILQCLCSITPLVEYFLSGKYITALQ--------
platypus_USP50         QGVTGLRNLGNTCYMNAVLQCLASIAPLVEYFLSGKYVTALH--------
rabbit_USP50           QGVTGLRNLGNTCYMNAILQCLGSISPLVEYFLSGKYITALQ--------
rat_USP50              QGVTGLRNLGNTCYMNAILQCLCSTSPLVEYFLSGKYITALQ--------
stickleback_USP50      -GVCGLDNSGNSCYLNAVLQCLCSTVPLVEHLLHRDTRKELG--------
tasmanian_devil_USP50  QGVTGLRNLGNTCYMNAILQCLCSITPLVEYFLSGKYITALQ--------
tetraodon_USP50        ----GLENSGNSCYLNAVLQCLCSTVPLVEHLLHPDTEAELA--------
wallaby_USP50          QGVTGLRNLGNTCYMNAILQCLCSITPLVEYFLSGKYITSLQN---F---
                           ** * **:* :**: *** :  **.: :   .    *

Ccow_USP50             ---KDCSEVATAFAYVMTDMWLGDSDCVSPEIFRSALGNLYPVFMKKTQQ
alpaca_USP50           LLPRDCSKVATAFAYVMTDMWLGDSDCVSPEILRSALGSLYPALTKKTQQ
anole_lizard_USP50     ---KDNGEIATAFAYLMNDMWLGDFDCVSPEVFRLVIGERYPAFIKKTQQ
bushbaby_USP50         ---KDCSEVATAFAYLMTDMWLGDSDCVSPEIFRSALGNLYPAFMKKTQQ
cat_USP50              LLPRDCSEVATAFAYVMTDMWLGDSECVSPEIFRSAFGNLYPAFMKKTQQ
```

```
chimpanzee_USP50        ---NDCSEVATAFAYLMTDMWLGDSDCVSPEIFWSALGNLYPAFTKKMQQ
dog_USP50               ---RDCREVATAFAYVMTDMWLGDSECVSPEVFRSALGSLYPAFMKKTQQ
dolphin_USP50           LLPRDCSEVATAFAYVMTDMWLGDSDCVSPEIFWSAFGKLYPAFMKKTQQ
elephant_USP50          ---KSCGEVATAFAYLMTDMWLGDSGCVSPEIFRSALGNLYPAFMKKTQQ
fugu_USP50              ---QSKCRLGGVFVQLLKKMWMGGYSSCAPVEARSVLGSILPQFNNYSQQ
guinea_pig_USP50        ---KDCREVATAFAYLLTDMWLGDADCVSPEEFRAALGRLHPDFTKRTQQ
human_USP50             ---NDCSEVATAFAYLMTDMWLGDSDCVSPEIFWSALGNLYPAFTKKMQQ
medaka_USP50            NSGRSKCRVSEVFVHLLEKMWMGSGSSCSPLEVRSALCSVLPQFNNDSQQ
megabat_USP50           LLPRDYSEVATAFAYVMTDMWLGDSDCVSPEIFRSALGNLYPALMKKTQQ
microbat_USP50          ---KDCSKVATAFAYVMTDMWLGDSDCVSPEILRSALGNLYPAFMKKTQQ
mouse_USP50             ---KDCSEVTTAFAYLMTDMWLGDSDCVSPEIFLSAVGSLYPAFLKKTQQ
opossum_USP50           ---KDSGEAATAFAYLMTDMWLGDSDCVSPEVFRTAIGKIYPTFLKRTQQ
platypus_USP50          ---RDRGEVATALAYLLTDMWLGDADCVAPEVFRLAVGDRHPAFGKKSQQ
rabbit_USP50            ---KDCSDVATAFAYLMTDMWLGDSDCVSPEIFRSALGNLYPAFMKKTQQ
rat_USP50               ---KDCSEVTTAFAYLMTDMWLGDSDCVSPEIFLSAVGSLYPAFLKKTQQ
stickleback_USP50       ---RSQCQVAQMFVRLLEEMWLGSGTSCAPVEARSVMCSVLPQFDNYSQQ
tasmanian_devil_USP50   ---KDSGEAATAFAYLMTDMWLGDSDCVSPEVFRAAIGKIYPAFLKRTQQ
tetraodon_USP50         ---RSKCRLAEVFARLLRKMWTGGCSSCAPLETRSVLSSILPQFNNYAQQ
wallaby_USP50           -LLRDCGEVATSFAYLMTDMWLGDSDCVSPEVFRSAIGKIYPAFLKRTQQ
                           ..       :. :: .** *.  . :*     ..    * : :  **

Ccow_USP50              DAQEFLIYVLNELHEALKKY--HQ---RKAYEKR------------SIPR
alpaca_USP50            DAQEFLIYVLNELHEALKKY--HR---RRSYEKK------------SLPR
anole_lizard_USP50      DAQEFLIHVLNELHEALKKT--SK---KRNQGTS------------SSTW
bushbaby_USP50          DAQEFLIYVLNELHEALKKTQYHR---RRSYEKR------------SIQR
cat_USP50               DAQEFLIYVLNELHESLKKC--HR----KSYEKG------------SIPR
chimpanzee_USP50        DAQEFLIC-VNELYEALKKYHYSR---RRPYEKG------------STQR
dog_USP50               DAQEFLIYVLNELHEALKKY--HR---KRSHEKG------------SVLR
dolphin_USP50           DAQEFLIYVLNELHETLKKY--HR---RRSYEKR------------SIPR
elephant_USP50          DAQEFLIYVLNDLHEALKKY--HR----RSYEKG------------STRR
fugu_USP50              DAQELLLFLLNVLHDDLKKVT--R---RQMRSSTLQQRR-K-----QGDK
guinea_pig_USP50        DAQEFLIYVLNALHEALSRYH--Q----RSGEKR------------STQR
human_USP50             DAQEFLICVLNELHEALKKYHYSR---RRSYEKG------------STQR
medaka_USP50            DAQELLLLLLNTLHDDFNKVL--L---NMFGRSTLCAQTDGVSGVKPARM
megabat_USP50           DAQEFLIYVLNELHEALKKY--HR---RRAYEKT------------SIPR
microbat_USP50          DAQEFLIYVLNELHESLKKF--HR---RRSWETG------------SVPR
mouse_USP50             DAQEFLIYVLNELHEALKKH--CR---RRVNEKR------------TGQS
opossum_USP50           DAQEFLIYVLNELHEALKKYR--R---KKPHEKV-------------TTK
platypus_USP50          DAQEFLIFVLNALHEALKKRRSRLRPGRKPSEKA-------------ASR
rabbit_USP50            DAQEFLIYVLNELHEALKKY--HR---RRSYEKG------------STQR
rat_USP50               DAQEFLIYVLNELHEALKKH--CR---GRASEKR------------TAQN
stickleback_USP50       DAQELMLFLLNALNDELKKVR--Q---HETRRASASRWH-R----VSGPV
tasmanian_devil_USP50   DAQEFLIYVLNELHEALKKYR--R---KKTQENG------------LSTK
tetraodon_USP50         DAQELLLFLLNLLHDDLKKMS--R---RRMRSSSRQQRQ-R-----RARR
wallaby_USP50           DAQEFLIYVLNELHEALKKFLFRR---KRPQEKG------------LTTK
                        ****:::  :* * : :.:
                        (ile136 in NP_987090.2, ile141 in Q70EL3)

Ccow_USP50              CCRKMIA-NESSIITRLFEGQLSYSIVCLKCENCTYKNEVFTIISLPIPS
alpaca_USP50            CCRKMIA-SESSIITRLFEGQLNYSIVCLKCENCTYKNEVFTVLSLPIPS
anole_lizard_USP50      DSRASYV-SESSIITRLFEGHLSYDIICLECQNTTYKNEIFTVLSLPIPY
bushbaby_USP50          CCRKVIA-NETSIITRLFEGQLNYSIVCLKCENCTYRNEVFTVLSLPIPC
cat_USP50               CCRKTIA-NEVSIITQLFEGQLNYSIMCLKCENCTYKNEVFTILCLPIPS
chimpanzee_USP50        CCRKWIT-TETSVITQLFEEQLNYSIVCLKCEKCTYKNEVFTVFSLPIPS
dog_USP50               CCRKVIA-SESSIITQLFEGQLNYSIMCLKCENCTYKNEVFTVLSLPIPS
dolphin_USP50           SCRKMIA-NESSIITRLFEGQLNYSIICLKCENCTYKNEVFTVLSLPIPS
elephant_USP50          CCRKVIA-TESSIITRLFEGQLNYDVICLKCENCSYKNEVFTVLSLPIPS
fugu_USP50              RSIAAAA-WETTAVSQLFEGQMSFMTLCMHCDHQAHSSQTFTVLSLPIPT
guinea_pig_USP50        CCSRGIA-SETSIITQLFEGQLNYSIMCLKCENCSYRNEVFTVLSLPIPS
human_USP50             CCRKWIT-TETSIITQLFEEQLNYSIVCLKCEKCTYKNEVFTVFSLPIPS
medaka_USP50            EQIKGCS-PHSNLVTRLFEGQLSYMCICMHCHHQAHNTQAFTVLSLPVPK
```

```
megabat_USP50          CCRKVTA-NESSIITRLFEGQLNYSIMCLKCENCTYKNEVFTILSLPIPS
microbat_USP50         CCRKAIA-SETSIVTRLFEGQLNYSIMCLKCENCTYRNEVFTVLPLPIPS
mouse_USP50            CCRKVPA-QETSIITRLFEGQLSYSITCLKCESCTHKNEVFTILSLPIPS
opossum_USP50          CYRKVAS-SESSIITRLFEGQLNYDIVCLKCENCTYKNEVFTVLSLPIPS
platypus_USP50         RGGKAAA-GESSIVSQLFEGQLSYDVVCLKCDSCSYKGETFTVLSLPIPS
rabbit_USP50           CCRKAIA-NETSIITQLFEGQLNYGIICLKCENCTYRNEVFTVLSLPIPS
rat_USP50              CCRKVPA-HETSIITRLFEGQLSYSITCLKCETCTYKNEVFTILSLPIPS
stickleback_USP50      CVCGAAATESTTMVSRLFEGQLGYTTLCTHCEHQTRSTQSFTVLSLPIPK
tasmanian_devil_USP50  CYRKVAS-SESSIITRLFEGQLNYDIVCLKCENCTYKNEVFTVLSLPIPS
tetraodon_USP50        GICADAA-WESTAVSLLFESQMSYVTVCMHCDHQTRSTQTFTVLSLPVPA
wallaby_USP50          CYRKVAS-SESSIITRLFEGQLNYDIVCLKCENCTYKNEVFTVLSLPIPS
                                  . :: *** ::.:   * .*.  :   : **:: **:*

Ccow_USP50             E-YECSLQDCLQCFFQQDTLTWNF----L-YSLF---LLEATVRAIISKV
alpaca_USP50           R-YECSLQDCLQCFFQQDTLTWNN----QIHCSLCETKQETAVRASISKA
anole_lizard_USP50     E-TECSLEIDIKMIFFLKGLDWLKGKPIQSLCSSGRIPQDSTGFETIVKL
bushbaby_USP50         E-YECSLQDCLQCFFQQDTLTWNN----QIRCAFCETNQETAVRASISKA
cat_USP50              D-YECSLQDCLQCFFQQDTLTWHN----QIHCSFCETKQETAVRASISKA
chimpanzee_USP50       K-YECSLRDCLQCFFQQDALTWNN----EIHCSFCETKQETAVRASISKA
dog_USP50              E-YECSLQDCLQCFFQQDTLTWNN----QIHCSFCETKQETAVRASISKA
dolphin_USP50          K-YECSLQDCLQCFFQQDTLAWDN----QIYCSFCETKQETAVKANISKA
elephant_USP50         E-YECSLQDCLQCFFQQDTLTWNN----QIHCSFCETKQETAVRASISKA
fugu_USP50             DTSKCTIEDCLSLFFQQTILTGGE----QMLCSVCGLRRETTVFTCLDNP
guinea_pig_USP50       E-YECTLQDCLQCFFQQDTLTWNN----QIHCSFCETKQETAVRASISKP
human_USP50            K-YECSLRDCLQCFFQQDALTWNN----EIHCSFCETKQETAVRASISKA
medaka_USP50           GNIKCSIQDCLALFFKQTVLTGGE----QAMCSVCGLKRETAIVTCVDRT
megabat_USP50          E-YECSLQDCLQCFFQQDTLTWNN----QIHCSFCETKQETAVRASISKA
microbat_USP50         E-YKCSLQDCLQCFFQQDTLTWNN----QIHCSFCETKQEAAVRASISKA
mouse_USP50            D-YECSLQDCLQCFFQQDTLTWSN----QIYCSFCEIKQEAAVRTTISKV
opossum_USP50          E-CECSLQECLGCFFQQDTLTWNN----QIHCAFCESKQDAAVRASIAKA
platypus_USP50         H-YQCSLQECLERFFQQDTLRWNN----QIYCSYCDAKQDAAVRATVVKA
rabbit_USP50           E-YECSLQDCLQCFFQQDTLTWNN----QIHCSFCETKQETAVRASISKA
rat_USP50              E-YECSLQDCLQCFFQQDTLTWNN----QIHCSFCEIKQETAVRTTISKA
stickleback_USP50      DTIKCSIQDCLSLFFGQTVLTAAE----QVLCSACGLKRETAVHTSLDKP
tasmanian_devil_USP50  E-CECSLQECLGCFFQQDTLTWNN----QIHCAFCESKQDAAVRASIAKA
tetraodon_USP50        EPVRCTVQDCLSLFFQQTVLAGGE----QMLCSACGLRRETAVFTSLDKP
wallaby_USP50          E-CECSLQECLGCFFQQDTLTWNN----QIHCAFCESKQDAAVRASIAKA
                          .*::.  :  :*    *           .       :::    : .

Ccow_USP50             PKTIVFHLKRFDILGTMKRKLRTD-IHYPLTNLDLTPYICPIFRK--HPK
alpaca_USP50           PKIIVFHLKRFDVLGTMKRKLRTD-IHYPLTNLDLTPYICPIFRK--HPK
anole_lizard_USP50     YSVCLIYNTELDTFGLYTKGLSIDSVNSPSAAASSSPHYTMNHSS--FIA
bushbaby_USP50         PKIIVFHLKRFDIQGMMKRKLRTN-IHYPLTNLDLTSYICPIFRK--HPK
cat_USP50              PKVIIFHLKRFDILGTMKKKLRTD-IHYPLTNLDLSPYICPVFRK--HPK
chimpanzee_USP50       PKIIIFHLKRFDIQGTTKRKLRTD-IHYPLTNLDLTPYICSIFRK--YPK
dog_USP50              PKIIIFHLKRFDILGTMKRKLRTD-IHYPLTNLDLTPYICPVFRK--HPK
dolphin_USP50          PKIIVF-LKRFDILGTMKRKLRTD-IHYPLTNLDLTPYICPIFRK--HPK
elephant_USP50         PKIIIFHLKRFDVQGTMKRKLRTH-IHYPLTNLDLTPYICPIFRK--HPK
fugu_USP50             PEILTLHLKRFGCKGKNQVKLRTN-VLFNM-KLNISPFLSSP-EQ--NSS
guinea_pig_USP50       PKTIIFHLKRFDIQGQVKRKLRTD-IHYPLTNLDLTPYICPVFRK--HPK
human_USP50            PKIIIFHLKRFDIQGTTKRKLRTD-IHYPLTNLDLTPYICSIFRK--YPK
medaka_USP50           PEILVLHLKRFGSKGKSQVKLRTN-VLFFM-KLDLSQFLSGLVPN--ESS
megabat_USP50          PKIMIFHLKRFDILGTTKRKLRTD-IHYPLTNLDLTPYICPIFRK--HPK
microbat_USP50         PKIMIFHLKRFDILGTMKRKLRTD-IHYPLTNLDLTPYICPVFRK--HPK
mouse_USP50            PKIIVFHFKRFDIQGTVKRKLRTD-IHYPLTNLDLTPYICPVFRK--HPM
opossum_USP50          PKTVIFHLKRFDCQGRMKRKLRTD-IHYPLNNLDLSPYIYPLFRK--HPK
platypus_USP50         PNVVIFHLKRFECYGKMKRKLRTN-IRYPLANLDLSPYIYPPCRK--HPK
rabbit_USP50           PKIIIFHLKRFDIQGTVKRKLRTD-IHYPLTNLDLTPYICPVFRK--HPK
rat_USP50              PKIIVLHLKRYDLGSTVERPGRSN-LHLPVRNLASCPYSCPDLRT--HVM
stickleback_USP50      PEILMLHLKRFSCKGRNQVKLRTN-VFFSS-RLNLSPFLSSSVQSTVYSS
tasmanian_devil_USP50  PKTVIFHLKRFDCQGRVKRKLRTD-IHYPLNNLDLSPYIYPLFRK--HPK
```

```
tetraodon_USP50        PEILALHLKRFGCRGKNQVKLRTN-VLFSM-KLSLSSFLSGPEQNSSCSS
wallaby_USP50          PKTVIXXXXXFDCQGRVKRKLRTD-IHYPLTNLDLSPYIYPLFRK--HPK
                        .            .        . :           .

Ccow_USP50             YNLCAVVNHFGDLDGGHYTAFSKNSVTQAWYSFDDTRVSEIPDTAVQTAA
alpaca_USP50           YNLCAVVNHFGDLDGGHYTAFCKNSVTQA---------------------
anole_lizard_USP50     LTTMFYENHFGDLDGGHYTAFCKHTLTQSWYSFDDSQISEISECDVHTSA
bushbaby_USP50         YNLCAVVNHFGDLDGGHYTAFCKNSVTQ----------------------
cat_USP50              YNLCAVVNHFGDLDGGHYTAFCKNSVTQA---------------------
chimpanzee_USP50       YNL--VVNHFGDLDGGHYTAFYKNSVTA----------------------
dog_USP50              YNLCAVVNHFGDLDGGHYTAFCKNSVTQAWYSFDDTRVSEIPDTSVQTAT
dolphin_USP50          YNLCAVVNHSGDLDGGHYTAFCKNSVTHA---------------------
elephant_USP50         YSLCAVVNHFGDLDGGHYTAFCRNSVTQAWYSFDDTRVSEIPNTSVQTAT
fugu_USP50             YSLYAVVNHTGNLNMGHYTALCQSTITGTWHHFDDSAVKEVQEDFVQSSS
guinea_pig_USP50       YNLCAVVNHFGDLDGGHYTASCRNSVTQAWYSFDDTRVSEIPAPLVQTSM
human_USP50            YNLCAVVNHFGDLDGGHYTAFCKNSVTQ----------------------
medaka_USP50           YHLYAVVNHTGHLNMGHYTALCYNSLAQTWHCFDDAAVCEVQEDRVQSPN
megabat_USP50          YNLCAVVNHFGDLDGGHYTAFCKNSVTQT---------------------
microbat_USP50         YNLCAVVNHFGDLDSGHYTAFCKNSVTQAWFSFDDTRVSEIPDTSVQTAT
mouse_USP50            YNLCAVVNHFGDLDGGHYTAFCKNSVTQAWYSFDDTRVSEIPDTSVQTAT
opossum_USP50          YSLCGVVNHFGDLDGGHYTAFCKNTVSQTWYSFDDTRVCEIPDSSVQTAA
platypus_USP50         YNLWAVVNHFGDLDGGHYTALCKNTVTQSWFSFDDTRVCEVPEAAVQTAA
rabbit_USP50           YNLCAVVNHFGDLDGGHYTAFCKNSVTQAWYSFDDTRVSEIPNTSVQTST
rat_USP50              VTFCSINNHFGDLDGGHYTAFCKNSVTQAWYSFDDTRVSEIPDTSVQTAT
stickleback_USP50      YRLYAVVNHTGHLNMGHYTALCHNACTRCWHCFDDSAVREVRDSLVQSPN
tasmanian_devil_USP50  YSLCGVVNHFGDLDGGHYTAFCKNTVNQTWYSFDDTRVCEIPDSAVQTAA
tetraodon_USP50        YSLYAVVNHTGNLNMGHYTALCLSTVTGTWHHFDDAAVREVQDESVQSSS
wallaby_USP50          YSLCGVVNHFGDLDGGHYTAFCKNTVNQT---------------------
                              ** *.*: *****    :         D

Ccow_USP50             AYLLFYSCQPFSIPTQKC----------------KT
alpaca_USP50           -----------------------------------W
anole_lizard_USP50     AYLLFYSSQTFSVPVKT------------------Q
bushbaby_USP50         -----------------------------------A
cat_USP50              -----------------------------------W
chimpanzee_USP50       -----------------------------------W
dog_USP50              AYLLFYSCQPFSIPIKKC----------------KS
dolphin_USP50          -----------------------------------W
elephant_USP50         AYLLFYSCQPFSI----------------------P
fugu_USP50             AYMLLYSRRLFQKPNIQG---------------LSV
guinea_pig_USP50       AYLLFYSWQPFSKATQK------------------C
human_USP50            -----------------------------------A
medaka_USP50           AYLLFY------------------------------
megabat_USP50          -----------------------------------W
microbat_USP50         AYLLFYSCQPFSIPIEK------------------C
mouse_USP50            AYLLFYSCQPFSIPAQKRKSQDSTPDHCKQAIRKWP
opossum_USP50          AYLLFYSCQPFSIPTPKCK--------------CES
platypus_USP50         AYLLCYSCQPFSVPSHG------------------C
rabbit_USP50           AYLLFYSCQPFSLPIQ-------------------K
rat_USP50              AYLLFYSCQPFSIPTQKRKSWDSTPDYGKPAVRKWP
stickleback_USP50      AYVLLYSRTPFQK----------------------P
tasmanian_devil_USP50  AYLLFYSCQPFSIPTPKYNYFSRISFFCKTIILSNP
tetraodon_USP50        AYMLLYSRKPVQKPNIH------------------G
wallaby_USP50          -----------------------------------W
```

Supplementary Figure 1. Alignment of USP50 protein sequences from various species. Ile-141 in human USP50 (Q70EL3) is highlighted in yellow.

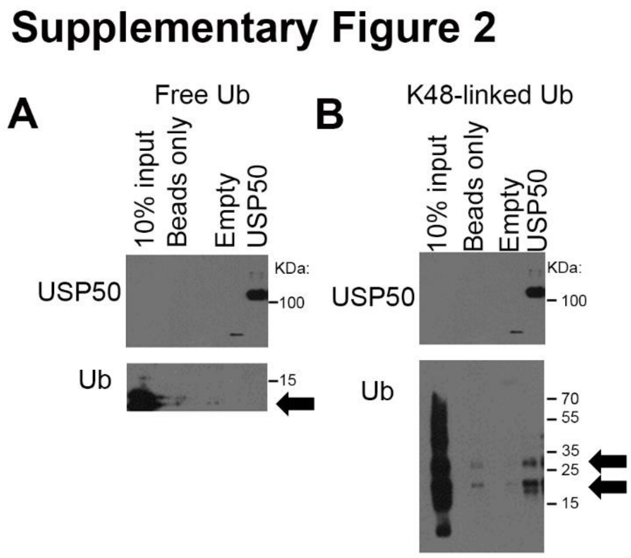

Supplementary Figure 2.

A. Amylose-bead precipitation alone (beads only), or precipitation of MBP (Empty), or MBP-USP50 fusion generated in bacteria and mixed with Ubiquitin (unconjugated). 10% of the Ub input was loaded in the lane on the left.
B. Amylose-bead precipitation alone (beads only), or precipitation of MBP only (Empty), or MBP-USP50 fusion generated in bacteria and mixed with K48-linked ubiquitin chains. 10% of the Ub input was loaded in the lane on the left.

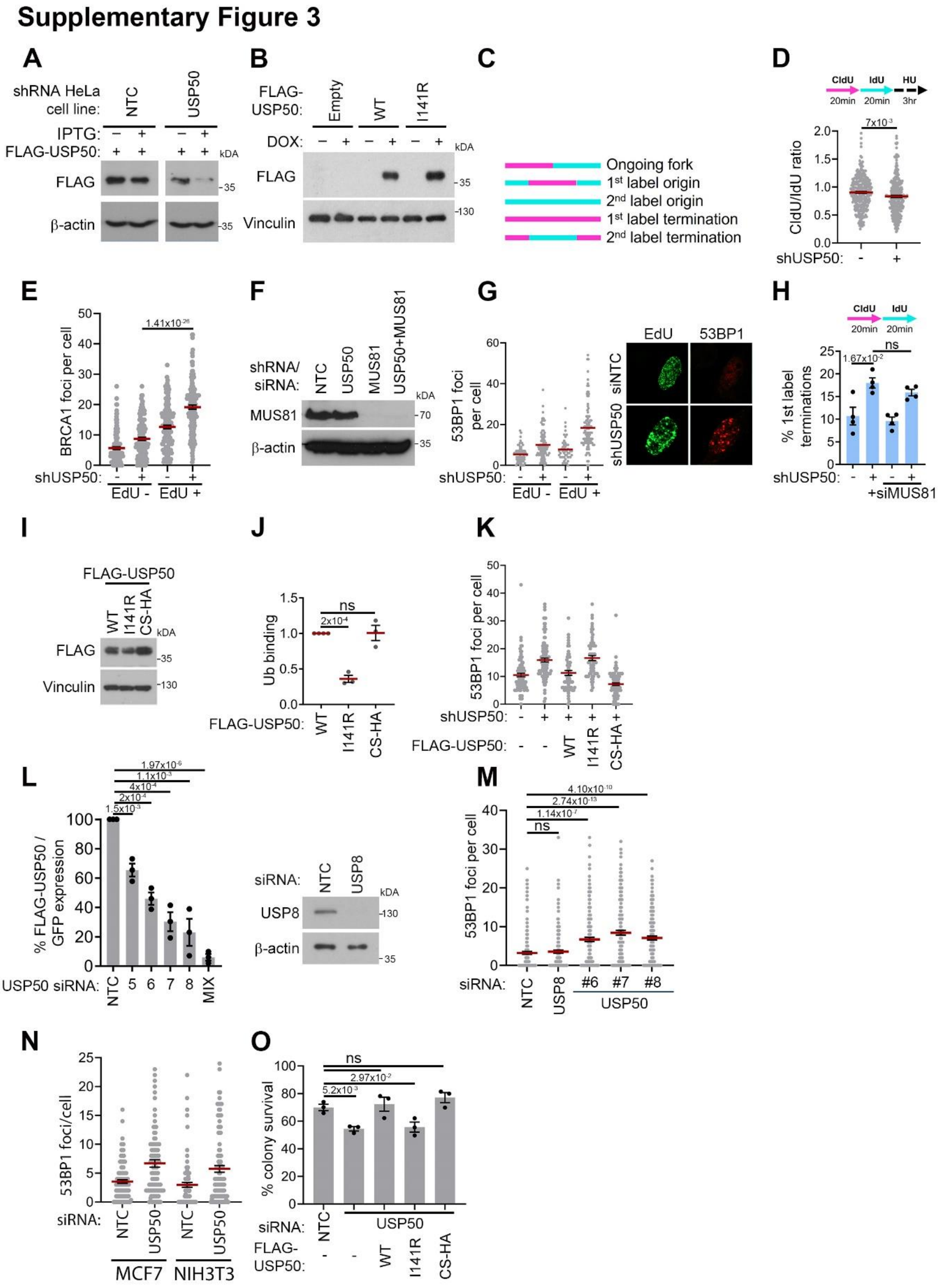

Supplementary Figure 3.

Where included, graphs indicate the mean ± SEM, exact P values are shown, and number of biological repeats is listed (n). All statistical analysis in this figure was performed using a two-tailed unpaired *t*-test. Source data are provided with this paper.

A. Immunoblot of two Hela cell lines transiently expressing wild-type FLAG-USP50, with and without IPTG to induce a non-targeting control sequence or shUSP50.

B. Immunoblot of Dox-inducible, shRNA-resistant FLAG-USP50 wild-type at the protein level (WT) or I141R HeLa cell lines treated, or not, with Dox. All cells were derived from cell line 2 (shown in A) and treated with IPTG to induce shUSP50.

C Diagram to illustrate structures identified in the DNA fibre assay to measure fork kinetics after sequential CldU and IdU labelling.

D HeLa cells, treated with siNTC (-) or shUSP50 (+), were incubated sequentially with CldU and IdU before 3 hours 5mM HU added. Tract lengths for each label were measured and the ratios determined. n=3, >200 fibres per condition.

E. BRCA1 foci numbers in EdU positive and negative cells treated with siNTC (-) or shUSP50 (+). n=3, >150 cells per condition.

F. Immunoblot of HeLa cells treated with siNTC, siUSP50, siMUS81 or a combination of both. MUS81 and β-actin levels (loading control) are shown.

G. 53BP1 foci numbers in EdU negative (-) and positive (+) HeLa cells treated with or without shUSP50. Representative images (right) and quantification (left). n=2, >46 cells per condition.

H. The % 1st label terminations were measured in HeLa cells treated with siNTC (-) or shUSP50 (+), with and without siMUS81. n=4, >200 fibres per condition.

I. Immunoblot of FLAG-USP50, I141R-FLAG-USP50 and CS-HA-FLAG-USP50 expression levels.

J. Immunoprecipitation of FLAG epitopes from HeLa cells expressing FLAG-USP50, I141R-FLAG-USP50 or CS-HA-FLAG-USP50 and Myc-Ub, probed for FLAG and Myc. Mean Myc-Ub normalised to both Myc-Ub and FLAG-USP50 expression in the whole cell lysate. n=3.

K. 53BP1 foci numbers in HeLa cells treated with siNTC (-) or shUSP50 (+) and complemented with FLAG-USP50, I141R-FLAG-USP50 or CS-HA-FLAG-USP50. n=2, >60 cells per condition.

L. Right: Immunoblot of USP8 protein levels in cells treated with siNTC or siUSP8 (β-actin loading control. Left: Quantification of non-siRNA resistant FLAG-USP50 expression, relative to GFP, in cells treated with siNTC, individual siUSP50 sequences (5-8) or a mix of all four. n=3.

M. 53BP1 foci numbers in HeLa cells treated with siNTC, siUSP8, or specific USP50 siRNAs. n=3, 200 cells per condition.

N. 53BP1 foci numbers in MCF7 and NIH3T3 cells treated with siNTC or siUSP50. n=2, >100 cells per condition.

O. Colony survival for shUSP50 expressing cells complemented with FLAG-USP50, I141R-FLAG-USP50 or CS-HA-FLAG-USP50 following 16 hours, 3 mM HU treatment. n=3.

Supplementary Figure 4

A

NTC (duplicates removed)

==

file name: rm_input.fasta
sequences: 29408
total length: 2213846 bp (2211777 bp excl N/X-runs)
GC level: 40.94 %
bases masked: 790945 bp (35.76 %)

==

	number of elements*	length occupied	percentage of sequence
SINEs:	2814	193181 bp	8.73 %
ALUs	2555	176796 bp	7.99 %
MIRs	258	16327 bp	0.74 %
LINEs:	3502	248156 bp	11.22 %
LINE1	3274	233823 bp	10.57 %
LINE2	205	12918 bp	0.58 %
L3/CR1	16	968 bp	0.04 %
LTR elements:	1749	121307 bp	5.48 %
ERVL	297	20245 bp	0.92 %
ERVL-MaLRs	732	49972 bp	2.26 %
ERV_classI	623	44050 bp	1.99 %
ERV_classII	87	6385 bp	0.29 %
DNA elements:	575	38494 bp	1.74 %
hAT-Charlie	221	14305 bp	0.65 %
TcMar-Tigger	247	17227 bp	0.78 %
Unclassified:	70	4004 bp	0.18 %
Total interspersed repeats:		605142 bp	27.36 %
Small RNA:	70	3949 bp	0.18 %
Satellites:	2037	149741 bp	6.77 %
Simple repeats:	675	27385 bp	1.24 %
Low complexity:	98	4728 bp	0.21 %

==

USP50 (duplicates removed)

==

file name: rm_input.fasta
sequences: 83172
total length: 3445086 bp (3442350 bp excl N/X-runs)
GC level: 42.61 %
bases masked: 570236 bp (16.57 %)

==

	number of elements*	length occupied	percentage of sequence
SINEs:	1975	105275 bp	3.06 %
ALUs	1863	99163 bp	2.88 %
MIRs	110	5967 bp	0.17 %
LINEs:	2400	130762 bp	3.80 %
LINE1	2292	124952 bp	3.63 %
LINE2	98	5302 bp	0.15 %
L3/CR1	6	311 bp	0.01 %
LTR elements:	1136	63094 bp	1.83 %
ERVL	164	9441 bp	0.27 %
ERVL-MaLRs	456	25229 bp	0.73 %
ERV_classI	456	25177 bp	0.73 %
ERV_classII	53	2861 bp	0.08 %
DNA elements:	327	18098 bp	0.53 %
hAT-Charlie	122	6764 bp	0.20 %
TcMar-Tigger	167	9182 bp	0.27 %
Unclassified:	44	2119 bp	0.06 %
Total interspersed repeats:		319348 bp	9.28 %
Small RNA:	262	12136 bp	0.35 %
Satellites:	3690	209776 bp	6.09 %
Simple repeats:	634	22913 bp	0.67 %
Low complexity:	113	6063 bp	0.18 %

==

B

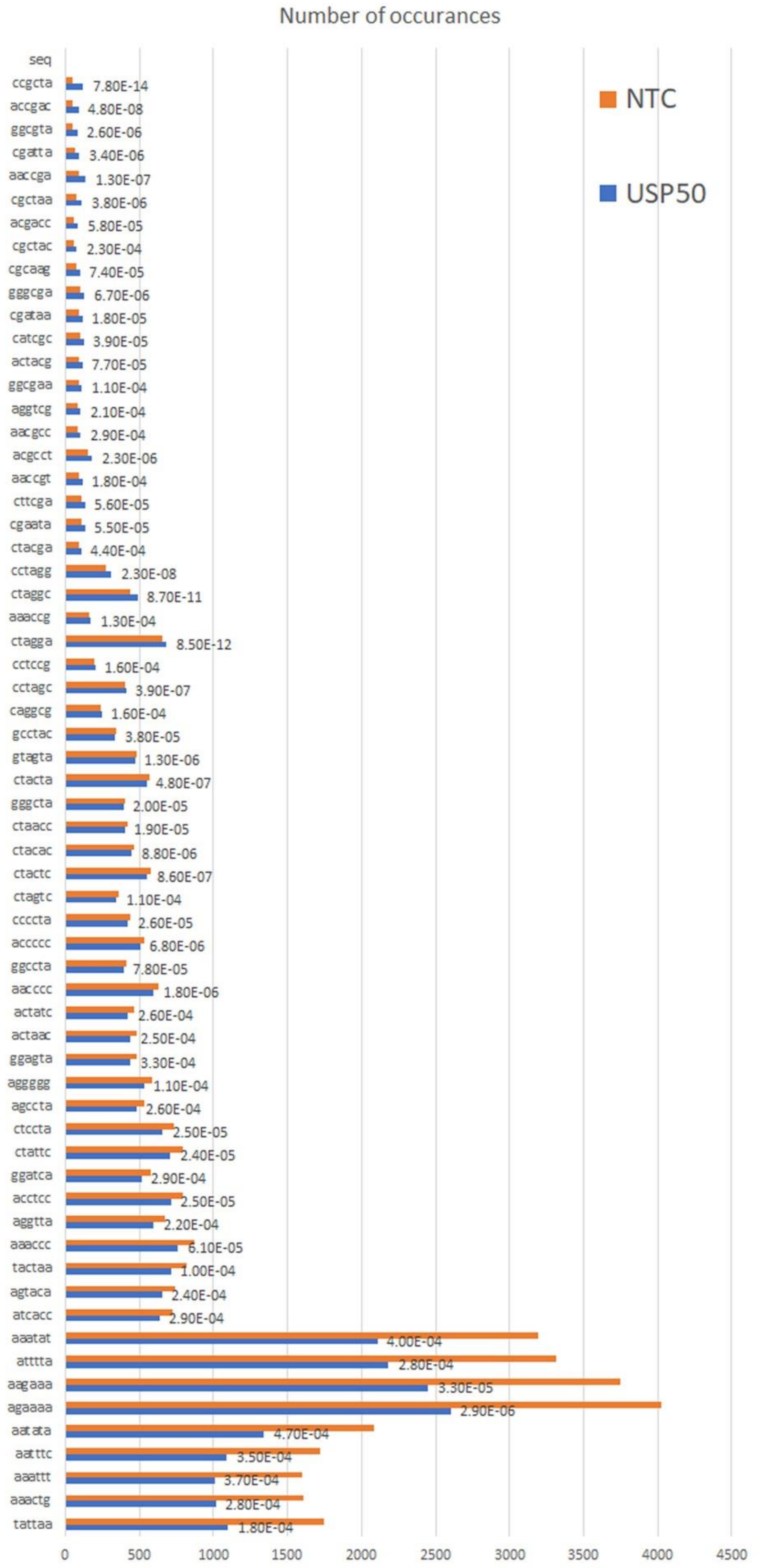
Number of occurances
NTC
USP50
seq
ccgcta 7.80E-14
accgac 4.80E-08
ggcgta 2.60E-06
cgatta 3.40E-06
aaccga 1.30E-07
cgctaa 3.80E-06
acgacc 5.80E-05
cgctac 2.30E-04
cgcaag 7.40E-05
gggcga 6.70E-06
cgataa 1.80E-05
catcgc 3.90E-05
actacg 7.70E-05
ggcgaa 1.10E-04
aggtcg 2.10E-04
aacgcc 2.90E-04
acgcct 2.30E-06
aaccgt 1.80E-04
cttcga 5.60E-05
cgaata 5.50E-05
ctacga 4.40E-04
cctagg 2.30E-08
ctaggc 8.70E-11
aaaccg 1.30E-04
ctagga 8.50E-12
cctccg 1.60E-04
cctagc 3.90E-07
caggcg 1.60E-04
gcctac 3.80E-05
gtagta 1.30E-06
ctacta 4.80E-07
gggcta 2.00E-05
ctaacc 1.90E-05
ctacac 8.80E-06
ctactc 8.60E-07
ctagtc 1.10E-04
ccccta 2.60E-05
accccc 6.80E-06
ggccta 7.80E-05
aacccc 1.80E-06
actatc 2.60E-04
actaac 2.50E-04
ggagta 3.30E-04
aggggg 1.10E-04
agccta 2.60E-04
ctccta 2.50E-05
ctattc 2.40E-05
ggatca 2.90E-04
acctcc 2.50E-05
aggtta 2.20E-04
aaaccc 6.10E-05
tactaa 1.00E-04
agtaca 2.40E-04
atcacc 2.90E-04
aaatat 4.00E-04
atttta 2.80E-04
aagaaa 3.30E-05
agaaaa 2.90E-06
aatata 4.70E-04
aatttc 3.50E-04
aaattt 3.70E-04
aaactg 2.80E-04
tattaa 1.80E-04
0
500
1000
1500
2000
2500
3000
3500
4000
4500

Supplementary Figure 4.

A. RepeatMasker statistics on sequences proximal to breaks in cells treated with siNTC or siRNA targeting USP50.

B. Frequency of significantly different occurrences of sequences (6 bp) near a double strand break site between shUSP50 (blue) and siNTC (orange) treated HeLa cells. Sequences are shown on the y-axis and the number of occurrences on the x-axis. Numbers on the right of the bars show the p-value. Source data are provided with this paper.

Supplementary Figure 5

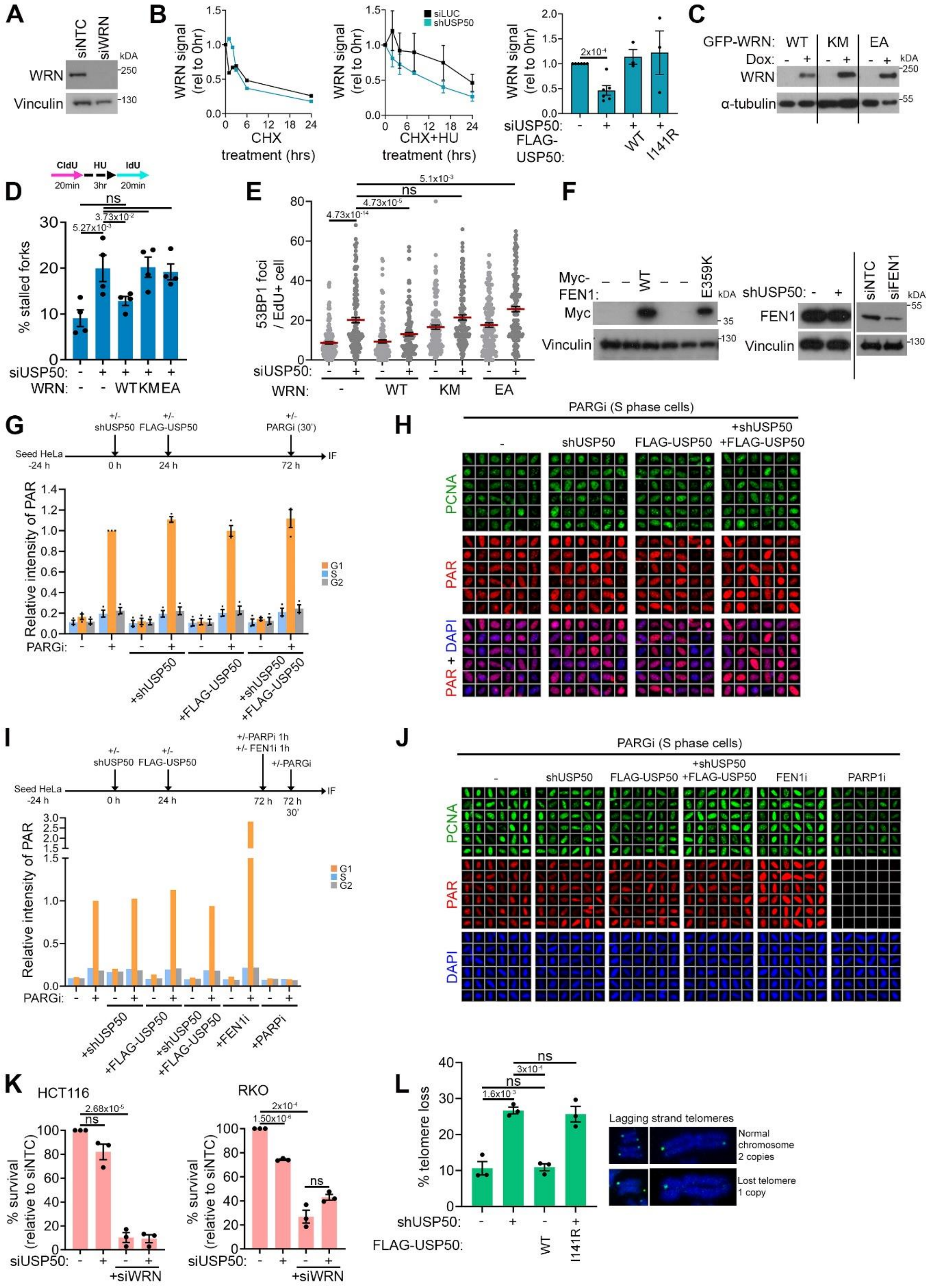

Supplementary Figure 5.

Where included, graphs indicate the mean ± SEM, exact P values are shown, and number of biological repeats is listed (n). All statistical analysis in this figure was performed using a two-tailed unpaired *t*-test. Source data are provided with this paper.

A. Western blot analysis of HeLa cells treated with siNTC or siWRN, blots are probed for WRN and Vinculin (loading control).

B. Left / Middle: quantification of WRN protein levels at various time points following cycloheximide treatment, with or without co-treatment of 3 mM HU. Right: quantification of WRN protein levels 16 hours after cycloheximide and HU treatment in cells expressing siNTC, shUSP50, FLAG-USP50 or I141R-USP50. n=1 (left), n=3 (middle), n=3 (right).

C. Western blot analysis of GFP-WRN HeLa variants, WT-WRN, K577M-WRN (KM), or E84A-WRN (EA) induced by Dox. Blots probed for WRN and α-tubulin (loading control).

D. The % of stalled forks from GFP-WRN HeLa variants, WT-WRN, KM, or EA induced by Dox and treated with siNTC (-) or siUSP50 (+). n=3, >200 fibres per condition.

E. 53BP1 foci per EdU positive cell in WT-WRN, KM, or EA expressing cells on a background of siNTC (-) or siUSP50 HeLa cells (+), treated with 5 mM HU for 3 hours. n=3, >120 cells per condition.

F. Left: western blot of siRNA resistant Myc-FEN variants; WT-FEN1, and E395K-FEN1 (EK). Blots were probed for Myc and vinculin (loading control). Middle: western blot of siNTC or shUSP50 expressing cells. Blots were probed for FEN1 and vinculin. Right: western blot of siNTC or siFEN1 treated cells. Blots were probed for FEN1 and Vinculin.

G. Top: illustration of the detection of PAR using the poly-ADP-ribose binding reagent MABE1031. Bottom: quantification of mean fluorescence intensities of PAR in cells ±shUSP50, ±FLAG-USP50, ±10 µM PARGi (for the last 20 mins). The mean intensity of PAR was normalised to the mean fluorescent intensity of S phase PAR in the presence of PARGi. n=3.

H. Representative ScanR images for G.

I. Top: illustration of the detection of PAR using the poly-ADP-ribose binding reagent MABE1031. Bottom: quantification of mean fluorescence intensities of PAR in cells ±shUSP50, ±FLAG-USP50, ±10 µM PARGi (for the last 20 mins). siNTC cells were incubated with 10 µM FEN1i (positive control) or 10 µM PARPi (negative control). n=1.

J. Representative ScanR images for I.

K. Colony survival of HCT116 (left) and RKO (right) cells treated with siNTC or siUSP50 ± siWRN. n=3.

L. Mean % of chromatids with lagging strand telomere loss after shUSP50 and complementation with WT or I141R FLAG-USP50. Representative CO-FISH image is shown (right) n=3, scored chromatid numbers per experiment: NTC: 1482, 956, 898; shUSP50: 1446, 652, 970; WT-USP50: 1118, 1512, 510; I141R-USP50: 490, 1430, 536). Note that the siNTC and shUSP50 datasets are shared with Figure 4H.

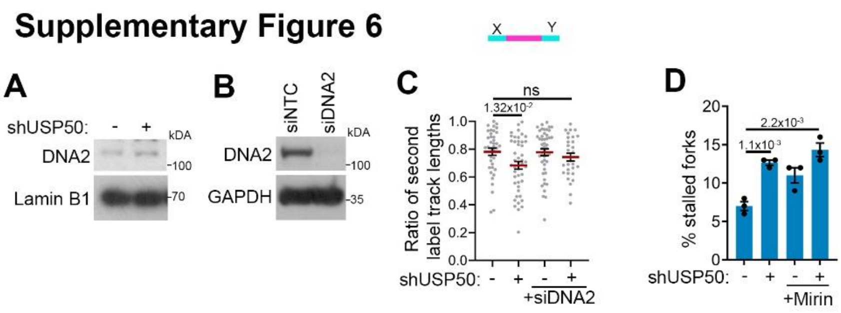

Supplementary Figure 6.

Where included, graphs indicate the mean ± SEM, exact P values are shown, and number of biological repeats is listed (n). All statistical analysis in this figure was performed using a two-tailed unpaired *t*-test.

A. Immunoblot of cells treated with siNTC or shUSP50. Blots were probed for DNA2 and Lamin B1 (loading control).

B. Immunoblot of cells treated with siNTC or siDNA2. Blots were probed for DNA2 and GAPDH (loading control).

C. Mean ratio of second-label tracts, either side of first labels after ±shUSP50 and ±co depletion ofDNA2. n=3, >29 first label origins measured.

D. Mean % of stalled forks from HeLa cells treated with siNTC or shUSP50, with and without co-treatment of 50 µM Mirin. n=3, >200 fibres per condition.

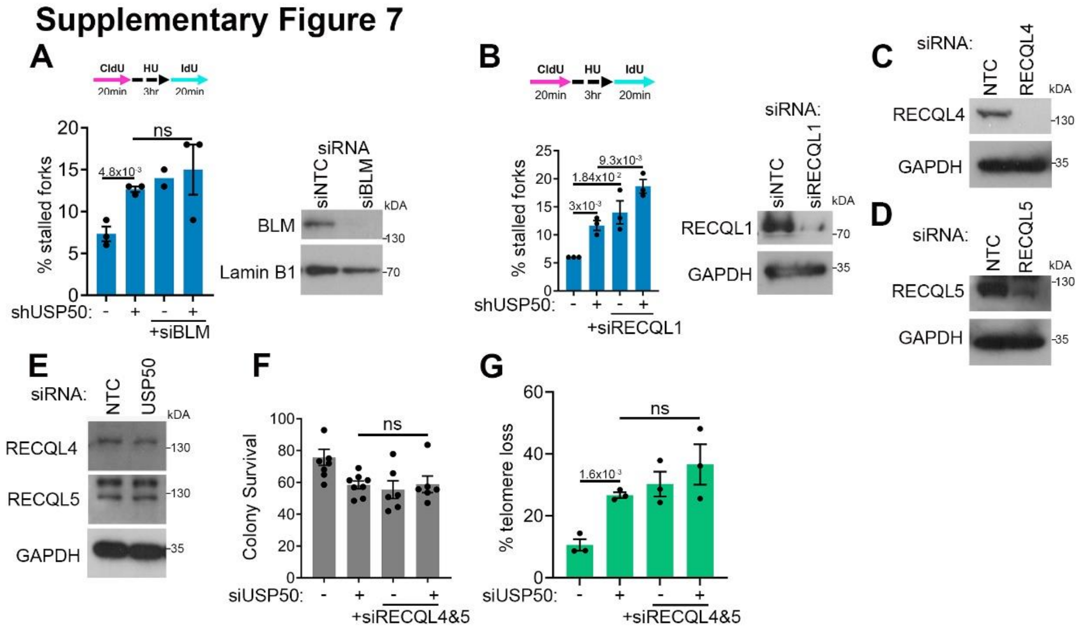

Supplementary Figure 7.

Where included, graphs indicate the mean ± SEM, exact P values are shown, and number of biological repeats is listed (n). All statistical analysis in this figure was performed using a two-tailed unpaired *t*-test. Source data are provided with this paper.

A. Left: mean % of stalled forks from HeLa cells treated with siNTC or shUSP50, with and without co-treatment of siBLM. n=3, >200 fibres per condition. Right: immunoblot of cells treated with siNTC or siBLM. Blots were probed for BLM and Lamin B1 (loading control).

B. Left: mean % of stalled forks from HeLa cells treated with siNTC or shUSP50, with and without co-treatment of siRECQL1. n=3, >200 fibres per condition. Right: immunoblot of cells treated with siNTC or siRECQL1. Blots were probed for RECQL1 and GAPDH (loading control).

C. Immunoblot of cells treated with siNTC or siRECQL4. Blots were probed for RECQL4 and GAPDH (loading control).

D. Immunoblot of cells treated with siNTC or siRECQL5. Blots were probed for RECQL5 and GAPDH (loading control).

E. Immunoblot of cells treated with siNTC or shUSP50. Blots were probed for RECQL4, RECQL5 and GAPDH (loading control).

F. Colony survival for shUSP50 expressing cells with and without co-depletion of RECQL4&5. following 16 hours, 1.25 mM HU treatment. n=6.

G. Mean % of chromatids with lagging strand telomere loss after shUSP50, with and without, co-depletion of RECQL4&5. n=3, scored chromatid numbers per experiment: NTC: 1482, 956, 898; shUSP50: 1446, 652, 970; siRecQL4/5: 178, 1708, 874; shUSP50 / siRecQL4/5: 374, 986, 810. Note that the siNTC and shUSP50 datasets are shared with Figure 4H.

Supplementary Table 1 – siRNA sequences

Target Gene	siRNA sequence
BLM exon 23	ACCGAAUCUCAAUGUACAUAG
BLM exon 7	GACGCUAGACAGAUAAGUUUA
DNA2	UACCGCUUAAAUCUAAGUCAA
FEN1 exon 2	GAUGCCUCUAUGAGCAUUUAU
MUS81 exon 1	ACGCGCUUCGUAUUUCAGA
MUS81 exon 4_5	GGAGCACCTGAATCCTAAT
MUS81 exon 16	AGTTGGTCCTCATCAAATA
NTC (luciferase)	CUUACGCUGAGUACUUCGA
RECQL1	GCCGCUUGGAAUAAAGAAGAU
RECQL4	ACCUCGAUUCCAUUAUCAUUU
RECQL5	GAGGAGAAGGUCCCUGUAAUU
USP50 #5	UAUGAUACCCUUCCAGUUA
USP50 #6	CAACACAUGCUGCGUGAAU
USP50 #7	CUACCCAGCAUUUACGAAA
USP50 #8	GGACCUCACUCCUUAUAUU
USP8	Dharmacon On-targetPLUS SMARTpool L-005203-00-0005
WRN exon 9	GAGGGUUUCUAUCUUACUA
WRN exon17	AUACGUAACUCCAGAAUAC

Supplementary Table 2 – Antibody details

Antibody	Animal	Assay	Dilution	Supplier	Cat number	Lot	RRID	Validation
53BP1	Rabbit	IF	1:2000	Abcam	ab36823	GR3360682-4	AB_722497	Tested and validated in IF. https://www.abcam.com/en-gb/products/primary-antibodies/53bp1-antibody-ab36823#
Biotin	Rabbit	PLA	1:50	Bethyl	A150-109A	N/A	AB_67327	Tested and validated https://www.fortislife.com/search?query=A150-109A&pageSize=5
BLM	Goat	WB	1:1000	Abcam	ab5446	GR3316091-1	AB_304894	Tested in immunoblot and IP. https://www.abcam.com/en-us/products/primary-antibodies/blooms-syndrome-protein-blm-antibody-ab5446
BrdU (CldU)	Rat	Fibres	1:1000	Abcam	ab6326	GR3173537-9	AB_305426	Validated in IF and ICC https://www.abcam.com/en-us/products/primary-antibodies/brdu-antibody-bu1-75-icr1-proliferation-marker-ab6326
BrdU (IdU)	Mouse	Fibres	1:750	BD Biosciences	347580	8151735	AB_400326	Validated in multiple formats: https://www.bdbiosciences.com/en-gb/products/reagents/flow-cytometry-reagents/clinical-discovery-

								research/single-color-antibodies-ruo-gmp/purified-mouse-anti-brdu.347580
DNA2	Rabbit	WB/IF	1:1000/1:200	Abcam	ab96488	1014291-2	AB_10677769	Validated inn WB: https://www.abcam.com/en-us/products/primary-antibodies/dna2-antibody-ab96488. Also see: Figure 4B and Supplementary Figure 6B herein.
Fc-fused anti-poly-ADP-ribose binding reagent	Rabbit	IF		Millipore	MABE1031		AB_2665467	https://www.merckmillipore.com/ES/es/product/Anti-poly-ADP-ribose-binding-reagent,MM_NF-MABE1031?ReferrerURL=https%3A%2F%2Fwww.google.com%2F
FEN1	Rabbit	WB/PLA	1:1000/1:200	Abcam	ab17994	ZC4249413C	AB_444168	Validated in IP, WB and IHCC https://www.abcam.com/en-us/products/primary-antibodies/fen1-antibody-ab17994. Also see Figure 3H and Supplementary Figure
FLAG (M2)	Mouse	WB/PLA	1:2000/1:300	Sigma	F1804	SLBT7654	AB_262044	https://www.sigmaaldrich.com/GB/en/product/sigma/f1804
GAPDH	Mouse	WB	1:10,000	Merck	CB1001	4049459	AB_2737054	https://www.merckmillipore.com/GB/en/product/Anti-GAPDH-Mouse-mAb-6C5,EMD_BIO-CB1001#anchor_PDS
GFP	Mouse	WB	1:5000	Roche	11814460001	47859600	AB_390913	https://www.antibodyregistry.org/AB_390913
γH2AX	Rabbit	IF	1:2000	Abcam	Ab2893	GR3242597-1	AB_303388	https://www.abcam.com/products/primary-antibodies/gamma-h2ax-phospho-s139-antibody-ab2893.html)
Histone H2B	Rabbit	WB	1:1000	Abcam	ab1790	GR3267844-1	AB_302612	https://www.abcam.com/en-gb/products/primary-antibodies/histone-h2b-antibody-chip-grade-ab1790
Histone H3	Rabbit	WB	1:1000	Abcam	ab1791	GR3446824-1	AB_302613	https://www.antibodyregistry.org/AB_302613
HUS1	Rabbit	PLA	1:500	Proteintech	11223-1-AP	1	AB_2248839	https://www.ptglab.com/products/HUS1-Antibody-11223-1-AP.htm
Lamin B1	Rabbit	WB	1:3000	Abcam	ab16048	953293	AB_443298	https://www.abcam.com/en-gb/products/primary-antibodies/lamin-b1-antibody-nuclear-envelope-marker-ab16048
Mus81	Mouse	WB	1:250	Novus	NB100-2064	CRT/18/64	AB_1109412	https://www.novusbio.com/products/mus81-antibody-mta30-2g10-3_nb100-2064

Myc	Mouse	WB	1:2000	Sigma	M5546	128M4898V	AB_260581	https://www.sigmaaldrich.com/GB/en/product/sigma/m5546
PCNA	Mouse	PLA	1:200	CST	2586	K3023	AB_2160343	https://www.cellsignal.com/products/primary-antibodies/pcna-pc10-mouse-mab/2586
Poly/mono-ADP Ribose	Rabbit	IF	1:1000	CST	#83732		AB_2749858	https://www.cellsignal.com/products/primary-antibodies/poly-mono-adp-ribose-e6f6a-rabbit-mab/83732
pRPA	Rabbit	WB	1:1000	Abcam	ab87277	GR3182765-41	AB_1952482	https://www.abcam.com/en-gb/products/primary-antibodies/rpa32-rpa2-phospho-s4-s8-antibody-ab87277
RECQL1	Rabbit	WB	1:3000	Fisher	PA5-27099	YC3851675E	AB_2544575	https://www.thermofisher.com/antibody/product/RecQ1-Antibody-Polyclonal/PA5-27099
RECQL4	Rabbit	WB	1:1000	Proteintech	17008-1-AP	00045772	AB_2238324	https://www.ptglab.com/products/RECQL4-Antibody-17008-1-AP.htm
RECQL5	Mouse	WB	1:1000	CST	5847	1	AB_10834807	https://www.cellsignal.com/products/primary-antibodies/recql5-1a2-mouse-mab/5847
RPA	Mouse	WB	1:3000	Millipore	NA13	GR3249141-1	AB_565121	https://www.merckmillipore.com/ES/es/product/Anti-Replication-Protein-A-Ab-1-Mouse-mAb-RPA70-9,EMD_BIO-NA13
Swine α Rabbit HRP	Swine	WB	1:5000	Dako	P0217	20047666	AB_2728719	https://www.agilent.com/store/productDetail.jsp?catalogId=P021702-2&catId=SubCat3ECS_244828
Tubulin	Mouse	WB	1:1000	Santa Cruz	sc-5286	H0613	AB_628411	https://www.scbt.com/p/alpha-tubulin-antibody-b-7
USP8	Sheep	WB	1:1000	R&Dsystems	AF7735	CHCT012007A	AB_2844099	https://www.rndsystems.com/products/human-usp8-antibody_af7735?gad_source=1&gclid=Cj0KCQjw2ou2BhCCARIsANAwM2EaiQ3ZAKyhDHsXH5vuyjEfKkjD1MLbB5ueeMLZfmUL2KfG37EJhekaAvGYEALw_wcB&gclsrc=aw.ds
Vinculin	Rabbit	WB	1:1000	Abcam	ab129002	1010179-37	AB_11144129	https://www.abcam.com/products/primary-antibodies/vinculin-antibody-epr8185-ab129002.html
WRN	Rabbit	WB/PLA	1:1000/1:200	Abcam	ab124673	08297-3C11	AB_10972871	https://www.abcam.com/en-us/products/primary-antibodies/werners-syndrome-helicase-wrn-antibody-epr6392-ab124673

WRN	Mouse	WB	1:1000	SLS	W0393	037M4751V	AB_1080598	https://www.scientificlabs.co.uk/product/antibodies/W0393-200UL
β-actin	Rabbit	WB	1:2000	Abcam	ab115777	GR3215935-1	AB_10899528	https://www.abcam.com/products/primary-antibodies/beta-actin-antibody-ab8227.html
Rabbit α Mouse HRP	Rabbit	WB	1:5000	Dako	p0161	20062080	AB_2687969	
Donkey α Rabbit AlexaFluor 488	Goat	IF	1:2000	ThermoFisher	A21206	1874771	AB_2535792	
Donkey α Mouse AlexaFluor 555	Goat	IF	1:2000	ThermoFisher	A31570	1774719	AB_2536180	
Donkey α Mouse AlexaFluor 488	Donkey	IF	1:2000	ThermoFisher	A21202	1975519	AB_141607	
Goat α Rat AlexaFluor 555	Goat	Fibres	1:2000	ThermoFisher	A21434	2626520	AB_141733	
Donkey α Sheep HRP	Donkey	WB	1:10000	Sigma	A3415.5ML	N/A	AB_258076	
Donkey α Rabbit AlexaFluor 555	Donkey	IF	1:2000	ThermoFisher	A31572	1945911	AB_162543	
AlexaFluor 647 azide	-	ClickIT	1:2000	ThermoFisher	A10277	2591116		

Supplementary Table 3 – Key Chemicals

Reagent	Source	Identifier
BrdU	Invitrogen	11594167
C5 DNA2i	Cambridge Biosciences	HY-128729-5mg
Exonuclease III	Promega	M1811
FEN1 inhibitor	Synthesized as described in [132].	Compound 24 in [132], Compound 1 in [133].
Hoechst 33258	Biotechne	**5824/50**
Hydroxyurea	Sigma Aldrich	**H8627**
Mirin	Alfa Aesar	J67462
PARG inhibitor (PDD 0017273)	Tocris/Sigma Aldrich	5952/SML1781
PARP inhibitor (Olaparib, AZD2281, Ku-0059436)	ApexBio	A4154
Pyridostatin hydrochloride	Sigma Aldrich	SML2690
RNase A	VWR	A2760.0100
Telomerase inhibitor BIBR1532	Cambridge Biosciences	CAY16608
