# Supplementary material for: USP50 suppresses alternative RecQ helicase use and deleterious DNA2 activity during replication
Source: Nat Commun. 2024 Sep 16;15:8102. doi: 10.1038/s41467-024-52250-4 (PMC11405836; doi:10.1038/s41467-024-52250-4)
Supplement: Supplementary file 3 — Reporting Summary [file 41467_2024_52250_MOESM3_ESM.pdf]

nature portfolio

Corresponding author(s): Jo Morris

Last updated by author(s): Aug 19, 2024

Reporting Summary

Nature Portfolio wishes to improve the reproducibility of the work that we publish. This form provides structure for consistency and transparency in reporting. For further information on Nature Portfolio policies, see our Editorial Policies and the Editorial Policy Checklist.

Statistics

For all statistical analyses, confirm that the following items are present in the figure legend, table legend, main text, or Methods section.

n/a	Confirmed	
☐	☒	The exact sample size (*n*) for each experimental group/condition, given as a discrete number and unit of measurement
☐	☒	A statement on whether measurements were taken from distinct samples or whether the same sample was measured repeatedly
☐	☒	The statistical test(s) used AND whether they are one- or two-sided<br>*Only common tests should be described solely by name; describe more complex techniques in the Methods section.*
☒	☐	A description of all covariates tested
☒	☐	A description of any assumptions or corrections, such as tests of normality and adjustment for multiple comparisons
☐	☒	A full description of the statistical parameters including central tendency (e.g. means) or other basic estimates (e.g. regression coefficient) AND variation (e.g. standard deviation) or associated estimates of uncertainty (e.g. confidence intervals)
☒	☐	For null hypothesis testing, the test statistic (e.g. *F*, *t*, *r*) with confidence intervals, effect sizes, degrees of freedom and *P* value noted<br>*Give P values as exact values whenever suitable.*
☒	☐	For Bayesian analysis, information on the choice of priors and Markov chain Monte Carlo settings
☒	☐	For hierarchical and complex designs, identification of the appropriate level for tests and full reporting of outcomes
☒	☐	Estimates of effect sizes (e.g. Cohen's *d*, Pearson's *r*), indicating how they were calculated

Our web collection on statistics for biologists contains articles on many of the points above.

Software and code

Policy information about availability of computer code

Data collection	All specific softwares used for data collection are detailed in materials and methods section
Data analysis	Data analysis was performed using Excel, Image J and GraphPad as described in methods.

For manuscripts utilizing custom algorithms or software that are central to the research but not yet described in published literature, software must be made available to editors and reviewers. We strongly encourage code deposition in a community repository (e.g. GitHub). See the Nature Portfolio guidelines for submitting code & software for further information.

Data

Policy information about availability of data

All manuscripts must include a data availability statement. This statement should provide the following information, where applicable:

- Accession codes, unique identifiers, or web links for publicly available datasets
- A description of any restrictions on data availability
- For clinical datasets or third party data, please ensure that the statement adheres to our policy

The datasets generated during this current study are available from the corresponding authors on reasonable request

Research involving human participants, their data, or biological material

Policy information about studies with human participants or human data. See also policy information about sex, gender (identity/presentation), and sexual orientation and race, ethnicity and racism.

Reporting on sex and gender	N/A
Reporting on race, ethnicity, or other socially relevant groupings	N/A
Population characteristics	N/A
Recruitment	N/A
Ethics oversight	N/A

Note that full information on the approval of the study protocol must also be provided in the manuscript.

Field-specific reporting

Please select the one below that is the best fit for your research. If you are not sure, read the appropriate sections before making your selection.

☒ Life sciences ☐ Behavioural & social sciences ☐ Ecological, evolutionary & environmental sciences

For a reference copy of the document with all sections, see nature.com/documents/nr-reporting-summary-flat.pdf

Life sciences study design

All studies must disclose on these points even when the disclosure is negative.

Sample size	No sample size calculation was performed
Data exclusions	No data was excluded from the analysis
Replication	All experiments were independently replicated and this is reported for each experiment in the corresponding figure legend
Randomization	Randomisation was not required as experiments were performed on matched cell lines
Blinding	No blinding was done

Reporting for specific materials, systems and methods

We require information from authors about some types of materials, experimental systems and methods used in many studies. Here, indicate whether each material, system or method listed is relevant to your study. If you are not sure if a list item applies to your research, read the appropriate section before selecting a response.

Materials & experimental systems

n/a	Involved in the study	
☐	☒	Antibodies
☐	☒	Eukaryotic cell lines
☒	☐	Palaeontology and archaeology
☒	☐	Animals and other organisms
☒	☐	Clinical data
☒	☐	Dual use research of concern
☒	☐	Plants

Methods

n/a	Involved in the study	
☒	☐	ChIP-seq
☒	☐	Flow cytometry
☒	☐	MRI-based neuroimaging

Antibodies

Antibodies used	53BP1, Abcam, ab36823<br>Biotin, Bethyl, A150-109A<br>BLM, Abcam, ab5446<br>BrdU (CldU), Abcam, ab6326

BrdU (IdU), BD Biosciences, 347580
DNA2, Abcam, ab96488
Fc-fused anti-poly-ADP-ribose binding reagent, Millipore, MABE1031
FEN1, Abcam, ab17994
FLAG (M2), Sigma, F1804
GFP, Roche, 11814460001
Hexa-Histidine, Sigma, H1029
Histone H2B, Abcam, ab1790
Histone H3, Abcam, ab1791
HUS1, Proteintech, 11223-1-AP
Lamin B1, Abcam, ab16048
Mus81, Novus, NB100-2064
Myc, Sigma, M5546
PCNA, CST, 2586
Poly/mono-ADP Ribose, CSTl, #83732
pRPA, Abcam, ab87277
RECQL1, Fisher, PA5-27099
RECQL4, Proteintech, 17008-1-AP
RECQL5, CST, 5847
RPA, Millipore, NA13
Swine α Rabbit HRP, Dako, P0217
Tubulin Santa, Cruz, sc-5286
USP8, R&Dsystems, AF7735
Vinculin, Abcam, ab129002
WRN, Abcam, ab124673
WRN, SLS, W0393
β-actin, Abcam, ab115777

Rabbit α Mouse HRP, Dako, p0161
Donkey α Rabbit AlexaFluor 488, ThermoFisher, A21206
Donkey α Mouse AlexaFluor 555, ThermoFisher, A31570
Donkey α Mouse AlexaFluor 488, ThermoFisher, A21202
Goat α Rat AlexaFluor 555, ThermoFisher, 21434
Donkey α Sheep HRP, Sigma, A3415
Donkey α Rabbit AlexaFluor555, ThermoFisher, A31572
AlexaFluor 647 azide, ThermoFisher, A10277

Validation

53BP1 Tested and validated in IF. https://www.abcam.com/en-gb/products/primary-antibodies/53bp1-antibody-ab36823#
Biotin Tested and validated https://www.fortislife.com/search?query=A150-109A&pageSize=5
BLM Tested in immunoblot and IP. https://www.abcam.com/en-us/products/primary-antibodies/blooms-syndrome-protein-blm-antibody-ab5446
BrdU (CldU) Validated in IF and ICC https://www.abcam.com/en-us/products/primary-antibodies/brdu-antibody-bu1-75-icr1-proliferation-marker-ab6326
BrdU (IdU) Validated in multiple formats: https://www.bdbiosciences.com/en-gb/products/reagents/flow-cytometry-reagents/clinical-discovery-research/single-color-antibodies-ruo-gmp/purified-mouse-anti-brdu.347580
DNA2 Validated inn WB: https://www.abcam.com/en-us/products/primary-antibodies/dna2-antibody-ab96488. Also see: Figure 4B and Supplemental Figure 6B herein.
Fc-fused anti-poly-ADP-ribose binding reagent https://www.merckmillipore.com/ES/es/product/Anti-poly-ADP-ribose-binding-reagent,MM_NF-MABE1031?ReferrerURL=https%3A%2F%2Fwww.google.com%2F
FEN1 Validated in IP, WB and IHCC https://www.abcam.com/en-us/products/primary-antibodies/fen1-antibody-ab17994. Also see Figure 3H and Supplemental Figure
FLAG (M2) https://www.sigmaaldrich.com/GB/en/product/sigma/f1804
GFP https://www.antibodyregistry.org/AB_390913
https://www.abcam.com/products/primary-antibodies/gamma-h2ax-phospho-s139-antibody-ab2893.html)
Hexa-Histidine https://www.sigmaaldrich.com/GB/en/product/sigma/h1029
Histone H2B https://www.abcam.com/en-gb/products/primary-antibodies/histone-h2b-antibody-chip-grade-ab1790
Histone H3 https://www.antibodyregistry.org/AB_302613
HUS1 https://www.ptglab.com/products/HUS1-Antibody-11223-1-AP.htm
Lamin B1 https://www.abcam.com/en-gb/products/primary-antibodies/lamin-b1-antibody-nuclear-envelope-marker-ab16048
Mus81 https://www.novusbio.com/products/mus81-antibody-mta30-2g10-3_nb100-2064
Myc https://www.sigmaaldrich.com/GB/en/product/sigma/m5546
PCNA https://www.cellsignal.com/products/primary-antibodies/pcna-pc10-mouse-mab/2586
Poly/mono-ADP Ribose https://www.cellsignal.com/products/primary-antibodies/poly-mono-adp-ribose-e6f6a-rabbit-mab/83732
pRPA https://www.abcam.com/en-gb/products/primary-antibodies/rpa32-rpa2-phospho-s4-s8-antibody-ab87277
RECQL1 https://www.thermofisher.com/antibody/product/RecQ1-Antibody-Polyclonal/PA5-27099
RECQL4 https://www.ptglab.com/products/RECQL4-Antibody-17008-1-AP.htm
RECQL5 https://www.cellsignal.com/products/primary-antibodies/recql5-1a2-mouse-mab/5847
RPA https://www.merckmillipore.com/ES/es/product/Anti-Replication-Protein-A-Ab-1-Mouse-mAb-RPA70-9,EMD_BIO-NA13
Swine α Rabbit HRP https://www.agilent.com/store/productDetail.jsp?catalogId=P021702-2&catId=SubCat3ECS_244828
Tubulin https://www.scbt.com/p/alpha-tubulin-antibody-b-7
USP8 https://www.rndsystems.com/products/human-usp8-antibody_af7735?gad_source=1&gclid=Cj0KCQjw2ou2BhCCARIsANAwM2EaiQ3ZAKyhDHsXH5vuyjEfKkjD1MLbB5ueeMLZfmUL2KfG37EJhekaAvGYEALw_wcB&gclsrc=aw.ds
Vinculin https://www.abcam.com/products/primary-antibodies/vinculin-antibody-epr8185-ab129002.html
WRN https://www.abcam.com/en-us/products/primary-antibodies/werners-syndrome-helicase-wrn-antibody-epr6392-ab124673

WRN https://www.scientificlabs.co.uk/product/antibodies/W0393-200UL
β-actin https://www.abcam.com/products/primary-antibodies/beta-actin-antibody-ab8227.html
Further: Antibody validation is shown for several commercial primary antibodies within the manuscript (Figure 3H, Figure 4B, Figure 5H and I, Supplemental Figure 1A,B F, L, Supplemental figure 5A C F, Supplemental Figure 6B. Supplemental Figure 7,A C, D) that are critical to the conclusions of the work (WRN, FEN1, DNA2, RECQL4, RECQL5, RECQL1 FLAG and MYC, GFP, USP8, MUS81).

Eukaryotic cell lines

Policy information about cell lines and Sex and Gender in Research

Cell line source(s)	FlpIn HeLa (human female), MCF7 (human female) and NIH3T3 (murine male) cells were from Morris stocks (from commercial or colleague sources). HCT116 (human male) and RKO (human unspecified sex) were a gift from Prof Andrew Beggs, University of Birmingham.
Authentication	None of these cell lines have been authenticated
Mycoplasma contamination	All cell lines tested negative for Mycoplasma contamination
Commonly misidentified lines (See ICLAC register)	No commonly misidentified cell lines were used

Plants

Seed stocks	N/A
Novel plant genotypes	N/A
Authentication	N/A
